# How Argentinian Consumers Perceive the Safety of Irradiated Foods

**DOI:** 10.3390/foods13233891

**Published:** 2024-12-02

**Authors:** Tiago Rusin, Anna Lucia Casañas Haasis Villavicencio, Wilma Maria Coelho Araújo, Cristiane Faiad

**Affiliations:** 1 Radiation Technology Center, Nuclear and Energy Research Institute, IPEN-CNEN, Cidade Universitária, São Paulo 05508-000, SP, Brazil; eng.rusin@gmail.com (T.R.); annalucia.villavicencio@gmail.com (A.L.C.H.V.); 2 College of Health Sciences, University of Brasília, Campus Darcy Ribeiro, Brasília 70910-900, DF, Brazil; 3 Department of Clinical Psychology, Institute of Psychology, University of Brasília, Campus Darcy Ribeiro, Brasília 70910-900, DF, Brazil; crisfaiad@gmail.com

**Keywords:** food irradiation, perception, awareness, consumer, cross-cultural adaptation

## Abstract

Food irradiation is a process used for various purposes, the main function of which is food safety. Although food irradiation has been used to ensure food safety, most consumers are unaware of the basic concepts of irradiation, misinterpreting information and showing a negative perception towards food treated with ionizing radiation. This research aimed to develop the cross-cultural adaptation and validation of the Awareness Scale on Consumption of Irradiated Foods (ASCIF) for the Argentine population and culture. The scale included 31 items covering 4 factors: safety of irradiated foods (S), concepts (C), labeling (L), and awareness (A), which were able to assess the Argentine population’s knowledge of irradiated foods. The total number of respondents was 500 and the data were collected by means of an electronic survey. Statistical tests were carried out which met the validity assumptions and confirmed the validity and consistency of the psychometric scale by means of confirmatory factor analysis (CFA), Cronbach’s alpha coefficient, and exploratory structural equation modelling (ESEM). Analysis of the results showed that the majority of consumers are unaware of the benefits of irradiated foods. It was found that the scale met the criteria for evidence of validity and consistency, proving to be an efficient tool for assessing potential challenges and opportunities in the Argentinian market for irradiated foods. The process was approved by the Research Ethics Committees of Brazil and Argentina and followed the adaptation methodologies of the International Test Commission (ITC) with processes of translations and retranslations and application of the scale in Argentina.

## 1. Introduction

The agricultural sector plays a strategic role in the economy and geopolitics of the countries involved in importing and exporting food inputs and products. Food preservation is a major challenge for the various stakeholders, such as academia, companies, and governments seeking hegemony and dominance in food technology. Food processing aims to maintain food safety in terms of physical, chemical, sensory and nutritional aspects, as well as extending the shelf life of food products, addressing issues of access and distance, among others [1]. The physical–chemical, microbiological, sensory, and nutritional parameters of raw materials, ingredients, food additives, processing aids, and technologies are governed by the Codex Alimentarius [2].

Conventional thermal preservation methods are still the most widely used in the food industry. These methods include pasteurization, sterilization, evaporation, refrigeration, freezing, and the control of osmotic pressure and pH levels, among others. The preservation and safety of food products is mainly based on reducing and preventing the growth of micro-organisms, whether pathogenic or not. This can include the reduction of some thermosensitive food ingredients, especially vitamins and polyphenols, which are directly linked to food quality [3,4].

Food irradiation is a technology used to promote food safety by controlling insect infestation, reducing the microbial load and eliminating pathogenic microorganisms. In addition, this process delays or eliminates natural biological processes, such as ripening and germination, in fresh food [5]. Irradiated food is food that has been subjected to the process of ionizing irradiation, with the aim of improving preservation and food safety. This process uses high-energy radiation, such as gamma rays, X-rays, or electron beams, to destroy microorganisms, insects, and parasites, without making the food radioactive [6].

The use of this technology has been recommended by the World Health Organization (WHO) since 1980 [7,8]. The Código Alimentario Argentino [9] regulates the use of radiation in food, establishing the authorized sources of radiation, the classes of food and the intended objectives, as well as the maximum dose limit (quilogray—kGy). Food irradiation is only justified when it meets a technological need or contributes to achieving a food hygiene objective and should not be used as a substitute for good manufacturing practices (GMP). The doses used must be appropriate to the intended technological and public health objectives and must comply with appropriate irradiation processing practices. The Código Alimentario Argentino [9] sets out the guidelines for consumer information, stipulating that the logo and the phrase “Food treated with ionizing energy” must be used.

In 2005, the global amount of food treated by irradiation was 0.41 million tons (M t), broken down as follows: 46% dehydrated spices and vegetable products, 20% grains and fruit, 8% meat and fish, 22% garlic, cabbage, and potatoes, and 4% others (mushrooms, honey, etc.). In the United States, Canada, and Brazil, 0.101 M t of spices, 0.007 M t of fruit, and 0.008 M t of meat were irradiated, totaling 0.116 M t (29%). The global export of irradiated food products is close to half a million tons, 40% of which goes to China, 20% to the USA, and 13% to Vietnam, followed by Mexico with 8%. Other countries together account for 19%, noting that within each country, the amount of irradiated food produced and marketed is much greater. In 2019, China, the world leader in food irradiation, had 130 multipurpose facilities with cobalt-60 sources, three-quarters of which were exclusively for food. In addition to these, there were also 78 commercial units with electron accelerators, with 5 to 10 new facilities expected to be built every year. China irradiated 0.6 M t and 1.0 M t of food for domestic consumption in 2015 and 2020, respectively [10,11,12].

Despite the benefits of food irradiation and the adoption of quality standards validated by the Codex Alimentarius Commission (CAC) by the industry, consumers are often unaware of the scientific basis behind these processes and misjudge the quality of products, giving rise to a mistaken perception generated by their lack of knowledge and/or understanding, as demonstrated in some studies [1,6,8,13,14,15,16,17].

Research carried out with Turkish consumers using a questionnaire to assess awareness and acceptance of irradiated foods, through the influence of benefit and price statements, showed that the majority (80%; n = 355) of the 444 participants were unsure about the safety of irradiated foods. The authors identified that when consumers were informed about the benefits of food irradiation, the level of positive attitude towards irradiated foods increased substantially (62%); they also observed that the intention to purchase irradiated foods was higher (44%) when the price of these products was equal to that of non-irradiated foods [15].

Another study carried out in Brazil with 218 people, using interviews as the data collection method, indicated that 59.6% (n = 130) of those interviewed did not know that irradiation is a food preservation process. For 16% (n = 35) of those interviewed, irradiated food meant the same as radioactive food; 62% (n = 135) said they did not know if irradiating food could harm the consumer’s health and/or the environment; 45% (n = 98) of those interviewed said they looked at food labels frequently, and the majority said that the quality attribute is what determines the purchase; 92% (n = 201) did not know the symbol of irradiation, the Radura. Of these 92%, 16% (n = 32) would buy irradiated food because of the influence of the symbol, even without knowing its meaning [18]. Consumers in Mexico City, for example, accepted irradiation as a technology to treat water used for irrigation since this process would eliminate the number of cases of foodborne diseases (FBDs) caused by contaminated fresh food, thus minimizing public health problems [16].

The use of the ASCIF in Shubayr’s research [1] presented an innovative approach to measuring public awareness of irradiated foods in Saudi Arabia. The results of the survey highlighted the need for educational initiatives aimed at improving the understanding and acceptance of irradiated foods, especially among different demographic groups [1]. When used in another culture, the ASCIF scale needs to undergo a process of cross-cultural adaptation and validation according to the methodology proposed by the ITC [19].

**Figure 1:**
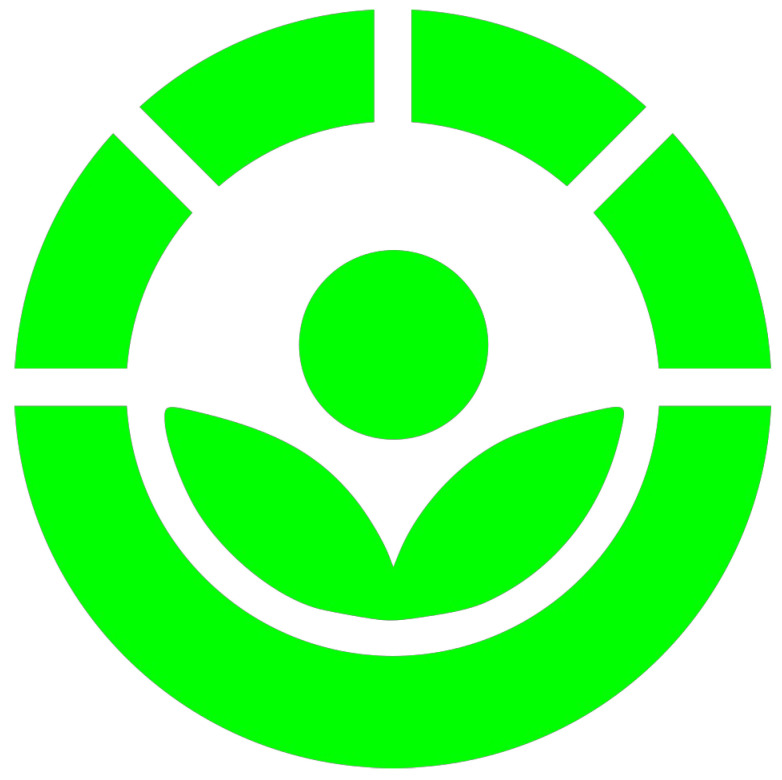
The international Radura logo.

Clear labeling, with the “Radura” symbol on the panel (Figure 1), is essential to inform, mitigate consumer fears, and increase confidence in irradiated foods [20].

The Codex General Standard for the Labelling of Prepacked Foods [21] requires that the label of a food that has been treated with ionizing radiation contain a written statement indicating this treatment in the immediate vicinity of the food’s name. The use of the international food irradiation symbol, Radura, is optional, but when it is used, it must be in close proximity to the name of the food. The legislation of many countries, such as the Código Alimentario Argentino [9], requires that the industry, when using irradiation in food, must ensure that the end customer is aware of this process, making this clear on the packaging.

While the presence of the Radura symbol is intended to inform consumers that a product has been irradiated, studies indicate that a substantial portion of the population remains unaware of this symbol. For instance, research shows that misconceptions about the safety and nutritional quality of irradiated foods are prevalent, often fueled by incorrect beliefs that such foods are radioactive or nutritionally depleted [1]. This lack of awareness can lead to misunderstandings about the safety of irradiated foods, as many consumers erroneously associate irradiation with harmful effects, such as radiation exposure or nutritional depletion [1,22].

Furthermore, the effectiveness of labeling is significantly influenced by consumer understanding and perceptions. A study conducted in China found that consumer acceptance of irradiated food is closely linked to their awareness and understanding of the technology; only 42% of respondents indicated they could accept having consumed irradiated food, while a notable percentage expressed reluctance due to misinformation and negative associations with the term “irradiation” [23]. Similarly, Italian consumers exhibited a lack of awareness regarding the benefits of food irradiation, often fearing that irradiated foods could be radioactive or contain harmful compounds [22]. This highlights a critical gap in consumer education and the need for more effective communication strategies to enhance understanding of irradiated foods.

Irradiation is a strategic process for ensuring the quality and shelf life of fresh and processed foods, yet it is underused and little known by consumers, requiring ongoing efforts to educate and dispel myths related to the process. Demographic and cultural characteristics, perception of risk, trust in the irradiated food industry, public opinion, scientific knowledge, benefits, costs, and availability of choice stand out among the factors that affect consumer acceptability [6,16].

Knowledge about irradiated foods and their acceptance by consumers is not unanimous, given the association they often make with radioactivity and health risks, as well as misconceptions about the safety and nutritional value of irradiated products [1,23]. Studies show that their acceptance is related to awareness and understanding of the process, as well as indicating that a significant proportion of consumers would be more willing to buy irradiated food if they were well informed about the benefits and safety of these products [23,24].

Social psychology studies the process by which a response is provoked by a stimulus (object or context) that mental mechanisms confer on the human social sphere. In this sense, some theories can estimate and explain, by means of specific scales or instruments, the conditions necessary for changes in behavior, attitudes, beliefs or emotions [25,26,27]. These theories have been applied to studies of consumer behavior, knowledge, understanding, perception, and attitudes in order to understand their reactions to the stimuli they receive. In general, they are based on the assumption that individuals make their decisions in an eminently rational way and systematically use the information that is available, considering the implications of their actions before deciding whether or not to behave in a certain way, mediating the attitude–behavior relationship [25]. It is a dynamic and continuous process in which personal, environmental, and behavioral factors influence each other [27].

Studies carried out to assess consumers’ knowledge, understanding, or perception of food-related attributes using psychometric scales are scarce. The principles of developing psychometric scales are based on three procedures: theoretical, empirical, and analytical (statistical). The scales must adapt items, such as desirability, simplicity, clarity, relevance, precision, variety, modality, typicality, credibility, amplitude, and balance, in order to enable the evaluation and validation of the data obtained [28].

Some prior examples of this research include a scale built to assess Brazilian consumers’ understanding of food classification (understanding the “level of processing” of food (ULPF)) according to the concepts of the Food Guide for the Brazilian Population (GAPB) [29]; a scale to assess knowledge and acceptance of processed foods (the Consumer Knowledge of Food Processing and Acceptance of Processed Food (CKAFP) scale) [30]; an online survey of 489 Italian consumers to assess the perceived safety of food technology and the role of food technology neophobia (FTN) in the perceived safety of consumers of food products of animal origin [31]; the Awareness Scale on Consumption of Irradiated Foods (ASCIF) to assess Brazilian consumers’ knowledge of irradiated foods [32]; the Knowledge, Attitudes, and Practices (KAP) scale to assess the risk of contracting foodborne diseases in a sample of the Sicilian population [33]; and the Nutrition and Health Claims (NHCs) scale to investigate Italian consumers’ knowledge of the real meaning of claims and their relationship to a healthy diet [34], among many others. The Awareness Scale on Consumption of Irradiated Foods (ASCIF) was used to estimate public awareness regarding the consumption of irradiated foods in Saudi Arabia. The results of the survey highlighted the need for educational initiatives aimed at improving the understanding and acceptance of irradiated foods, especially among different demographic groups [1]. When used in another culture, the ASCIF scale needs to undergo a process of cross-cultural adaptation and validation according to the methodology proposed by the ITC [1,19].

Knowledge is the result of experience, and it is regarded as the sum of human cognitive experience. It refers to the depth and breadth of information learned and skills that are valued by the culture. It reflects the degree to which an individual has acquired useful knowledge and mastered valuable skills. On the other hand, domain-specific knowledge refers to the depth, breadth and mastery of specialized knowledge (the knowledge that not all members of a society may have). Specialized knowledge is usually acquired through work, leisure, or another interest [35,36,37]. Perception involves recognizing and interpreting sensory stimuli. Perception is closely associated with attention processes in that perception is the ability to make sense of the surrounding environment, while attention is the ability to concentrate on the perceived stimuli [38].

According to the Database of Industrial Irradiation Facilities (DIIF) [39], in Argentina, there are two facilities that provide food irradiation services: the multipurpose plant of the Comisión Nacional de Energía Atómica (CNEA) in Centro de Ezeiza and the industrial plant IONICS S.A., a private company operating in Tigre, Buenos Aires province. Therefore, consumer awareness of food safety is fundamental in Argentina, as misinformation and lack of knowledge can lead to hesitation in accepting new food technologies, such as irradiation.

Providing positive information about food irradiation can significantly increase consumer acceptance [40]. In Argentina, awareness campaigns have resulted in a notable increase in acceptance of irradiated foods [41]. This suggests that enhancing consumer education about the benefits and safety of irradiated foods can play a crucial role in improving food safety perceptions and practices.

The importance of irradiated foods in ensuring food safety needs to be highlighted, especially in a country, like Argentina, where foodborne illnesses represent a significant public health challenge. The irradiation process effectively reduces microbial load and extends shelf life, which is particularly beneficial in a region where food safety regulations may not be as stringent as in higher-income countries [10].

Furthermore, the lack of awareness about food safety practices among consumers can exacerbate the risks associated with foodborne illnesses, making it essential to implement educational initiatives that inform the public about safe food handling and the advantages of food irradiation [42]. The focus on Argentina offers a unique opportunity to address the intersection between food safety, consumer awareness and trends in irradiated food acceptance. Therefore, it is critical to promote awareness of irradiated foods as a viable solution to improve food safety and public health outcomes in the region.

Based on the hypothesis that Argentina is a country that uses irradiation as a method of preserving food and that no research has been found on the subject in the literature, and that Argentine consumers may be unconsciously consuming irradiated foods due to factors, such as a lack of knowledge about these products, or insufficient information on food labels, and doubts about the guaranteed safety of irradiated foods, the objective of this study is to carry out the cross-cultural adaptation and validation of the Awareness Scale on Consumption of Irradiated Foods (ASCIF). This is because this scale (ASCIF) showed good evidence of validity, and additional studies with different population profiles, social and cultural classes should be carried out to confirm the generalizability of the results. In this scale, 4 factors were found, namely safety of irradiated foods (S), concepts (C), labeling (L), and awareness (A), which represent the 31 items of the instrument, with good internal reliability indices [32].

## 2. Methodology

The cross-cultural adaptation of ASCIF followed the methodology proposed by the International Test Commission [19], with some adaptations based on the recommendations of literature [43].

### 2.1. Cross-Cultural Adaptation

The cross-cultural adaptation of instruments—involving both translation and cultural adaptation—is extremely important for the standardization of uniform assessments for globalization and comparison of their results, as well as for the development of multicenter research [44]. The International Test Commission [19] presents a well-established methodology for cross-cultural adaptation of psychometric scale. According to the ITC, translating the test is only one part of the part of the adaptation process. Adaptation is the broader term and refers to moving a test from one language and culture to another. Test adaptation refers to all activities, including deciding whether a test in a second language or culture could measure the same construct as in the first language; selecting translators; choosing a design to evaluate the work of test translators (e.g., translations and reverse translations); choosing any necessary accommodations; modifying the test format; conducting the translation; checking the equivalence of the test in the second language or culture; and performing other necessary validity studies. The ITC methodology (presents 18 guidelines organized into six categories: pre-condition (3), test development (5), confirmation [empirical analyses] (4), administration (2), score scales and interpretation (2), and documentation (2) [19].

The first session, called “Pre-Condition”, highlights the fact that decisions have to be made before the translation/adaptation process begins. The second session, “Test Development Guidelines”, focuses on the actual process of adapting a test. The third session, “Confirmation”, includes the guidelines associated with the compilation of empirical evidence to address the equivalence, reliability, and validity of a test in multiple languages and cultures. The final three sections are related to “Administration”, “Score Scales and Interpretation”, and “Documentation” [19].

### 2.2. Sample

According to the International Test Commission (ITC) methodology, the sample was divided into two groups. The first group was made up of five Argentinian language course professionals (two for translation, two for retranslation, and one to assess agreement between the original items and the retranslations) with knowledge of the Spanish language and knowledge of Argentine culture. The translations and retranslations were conducted independently, i.e., this evaluation was conducted in a “blind” manner, to guarantee the originality of the translation/retranslation process. The participants received the evaluation form for the proposed items together with the informed consent form, which was dated and signed for the validity of the analysis [19].

The second group was made up of Argentinian men and women from different social classes and levels of education, from the province of Buenos Aires, identified on the lists of employees of the Comisión Nacional de Energía Atómica (CNEA), universities (professors and students), companies in the food sector, and the Argentine population in general, as the instrument aims to reach a lay audience on the subject of irradiated foods, so social variability in the sample is desirable. Participants accepted the terms of the informed consent form before starting the survey by clicking next/continue in the collection system [19].

Respondents from all school levels (excluding the illiterate because the questionnaire requires their reading and comprehension) were considered for the research, with ages ranging from 18 to 70 years of age, of both sexes and of different social classes, with social variability being desirable in the sample. The instrument was applied online to these groups between 25 October 2023 and 7 December 2023, and the total number of respondents was 500.

### 2.3. Data Collection

The data were collected by electronic means, through the site https://pt.surveymonkey.com/ (accessed between 25 October 2023 and 7 December 2023). In order to evaluate the knowledge and behavior of consumers as well as the knowledge and habits of consumers on irradiated foods, a questionnaire with a Likert scale of agreement was used. The scale offered five options of answer for each affirmative, including strongly disagree (1), disagree (2), neither agree nor disagree (3), agree (4), and strongly agree (5). This instrument consisted of items that identified the socio demographic characteristics of the sample, behavior measures, and measures of knowledge about the subject.

To ensure that the collection was restricted to Argentina, the internet protocol (IP) range for access to the instrument’s collection link was limited to residents of the Province of Buenos Aires.

### 2.4. Statistical Treatment

The statistical treatments guided the analysis and validation of the instrument, providing the results and solution strategies for the problems identified.

Confirmatory factor analysis (CFA) provided evidence of validity, assessing the psychometric quality of the ASCIF scale. The reliability of the instrument was assessed on the basis of internal consistency, obtained using Cronbach’s alpha. These statistical analyses were carried out using IBM SPSS (version 21). As a complement, exploratory structural equation modelling (ESEM) was carried out to evaluate the factor structure and the StdYX coefficients, and this analysis was carried out using MPlus software (version 7).

### 2.5. Approval by Research Ethics Committees

This research project was approved in Brazil by the Research Ethics Committee of Brazil University on 20 October 2023 (Certificate of Presentation for Ethical Appreciation 74513223.3.0000.5494). It was also approved in Argentina by the Comité de Ética Central del Ministerio de Salud de la Provincia de Buenos Aires, on 22 September 2023 (Ethical Evaluation Report No. ACTA-2023-40111123-GDEBA-CECMSALGP).

## 3. Results and Discussion

### 3.1. Demographics

A total of 500 Argentine consumers (51% women, 49% men) were involved in the study to validate the scale. A total of 28.4% were aged between 30 and 39, and 47.8% said they had a university degree and an average family income of 2 to 5 minimum wages, equivalent to USD 900 to 2250 a month (24%). In addition, 59.8% reported having a partner, 28% were private-sector employees, and 27.6% students. Furthermore, 97% were native Argentines, 37.4% reported living with up to two people in the same house, 77.6% were responsible for food shopping at home, and 91% reported living in the Province of Buenos Aires (Table 1). Further details can be found in the database (S1.sav).

### 3.2. Adaptation of Awareness Scale on Consumption of Irradiated Foods (ASCIF)

To conduct this stage of the research, five professionals from Argentine language courses with knowledge of the Spanish language and Argentine culture were invited, as suggested by the ITC methodology [19]. Initially, each of the translators was asked to read and sign the informed consent form. The instrument was then translated.

The process consisted of presenting a form with the original version of the ASCIF and space for the translation into Argentinian Spanish. This form was handled independently by two translators. Once the first stage of translation had been completed by the two translators, two new forms were drawn up with the ASCIF versions translated into Spanish. These new forms were retranslated into Portuguese by two other volunteers. As a result, a fifth translator and the author of ASCIF concluded with a comparison between versions and Argentine culture, in order to arrive at the final version of ASCIF for Argentina (Table 1).

It is important to note that the translators had no contact, and that the translations and retranslations were carried out independently, i.e., this evaluation was carried out “blindly”, to guarantee the originality of the translation/retranslation process. Table 2 shows the evaluation of the translations/retranslations with the semantic equivalence between the original Brazilian version and the Argentine version of the ASCIF.

In order to comply with current legislation on food irradiation in Argentina, it was necessary to update item 21 of the instrument, in accordance with Article 174, chapter III: food products, of the Código Alimentario Argentino [9], with the following label information: “Food Treated with Ionizing Energy” (Table 2).

### 3.3. Confirmatory Analysis of Data

#### 3.3.1. Analysis of Assumptions

The statistical assumptions of normality, linearity, and singularity were analyzed. It was observed that the histograms, skewness and kurtosis values and significance tests (Shapiro–Wilk) indicated a normal distribution. Deviations from multivariate normality are innocuous when all the variables meet this condition [45]. The model was assumed to meet the linearity hypothesis. This was achieved by analyzing the residuals and observing that the points were randomly distributed around zero. At the same time, the uniqueness assumption was analyzed by the variables meeting the VIF criterion of less than 5 and tolerance greater than 0.1, as recommended by Hair et al. [45].

#### 3.3.2. Confirmatory Factorial Analysis

It can be seen from the CFA results (Table 3) that the factor loadings remained at *p* < 0.05. For the safety of irradiated foods factor (S), they ranged from 0.855 (0.013) to 0.954 (0.004); for the concepts factor (C), they ranged from 0.848 (0.006)–0.947 (0. 005); for the labeling factor (L), they ranged from 0.837 (0.017)–0.956 (0.006); and for the awareness factor (A) they ranged from 0.820 (0.017)–0.859 (0.012).

We can see that question 8 (Q8. I consciously consume irradiated food) had the lowest ASCIF score of 2.396 and question 16 (Q16. I consider it necessary to carry out educational campaigns to inform the population about the irradiation of food) had the highest score of 3.884 (Table 3).

In order to observe the correlation between the factors, the main factors (PAF) were extracted and rotated using Oblimin rotation. Table 4 shows the correlations of ASCIF magnitude. There was a high correlation between the factors S and C (0.711), S and L (0.627), and C and L (0.438); on the other hand, there was a low correlation between S and A (−0.738), C and A (−0.650), and L and A (−0.476).

**Figure 2:**
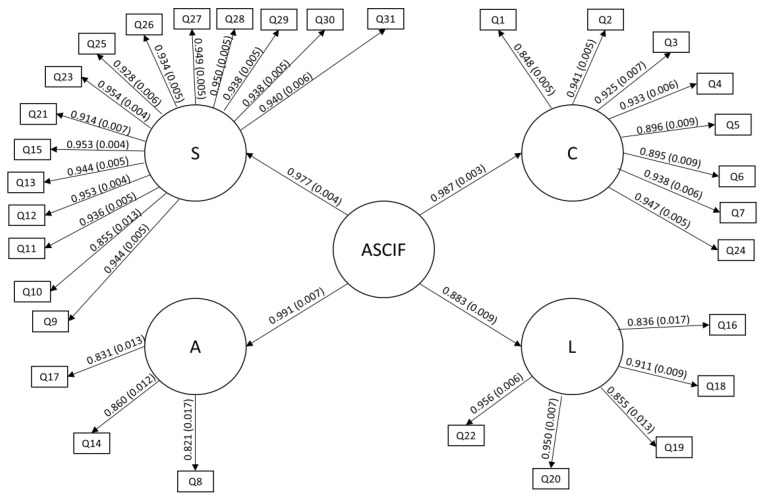
ASCIF second-order model.

Figure 2 shows the final structure of the second-order ASCIF model with its items (31) distributed among the four factors (S, C, L, and A) with the respective factor loadings and s.e. for the theoretical model. As can be seen, the ASCIF shows strong factor loadings with the model, indicating an instrument that is adherent to the reality of the Argentine population.

Renno and Wood point out that CFA can treat factors as latent variables, allowing for a more nuanced assessment of convergent and discriminant validity [46]. This approach is particularly useful in complex models, such as ASCIF. Similarly, Langdridge et al. used CFA to analyze the relationships between variables, reinforcing the importance of these statistical techniques in establishing discriminant validity [47]. In this sense, the establishment of discriminant validity was essential to guarantee the integrity of the ASCIF measures, providing evidence of the instrument’s validity.

#### 3.3.3. Reliability

Cronbach’s alpha index was used to test the internal consistency of the factors. The alpha index for the model was 0.988. According to Taber [48], the Cronbach’s alpha value found can be classified as excellent.

#### 3.3.4. Exploratory Structural Equation Modelling (ESEM)

**Figure 3:**
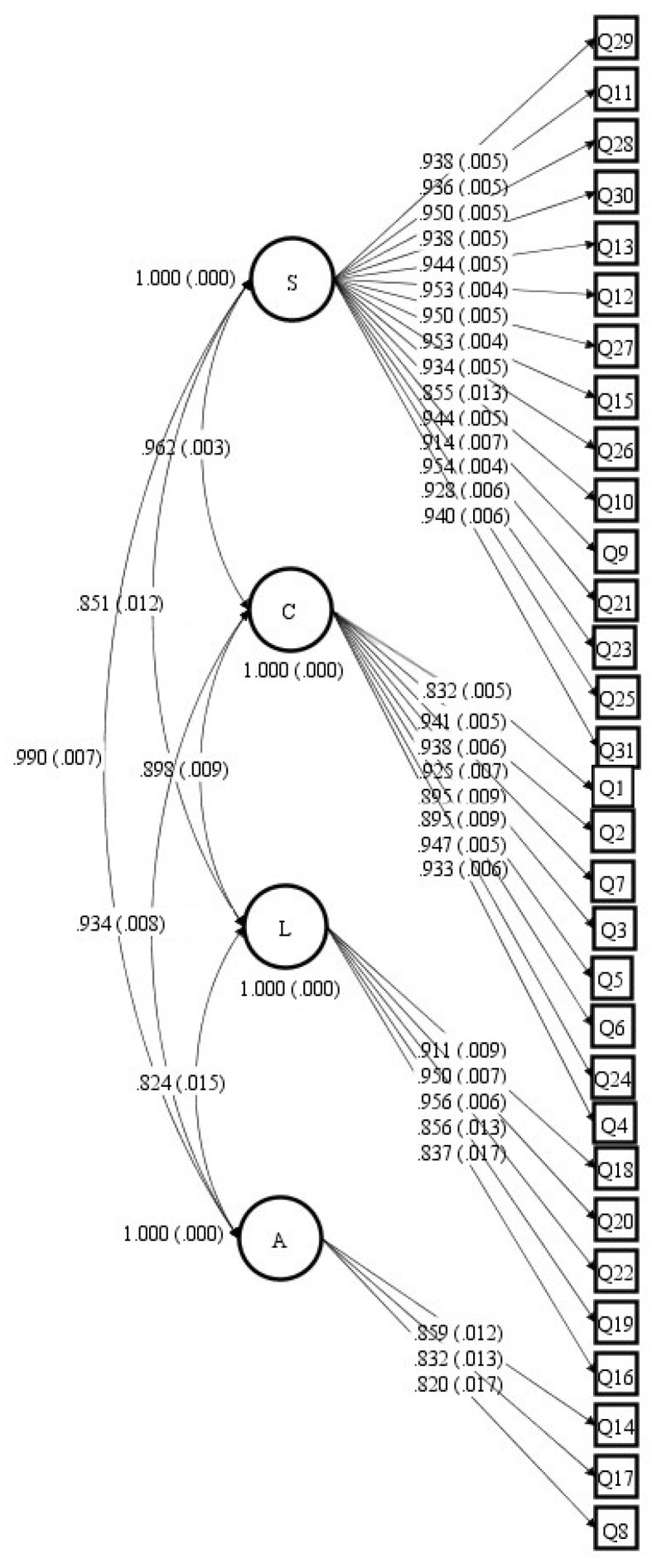
ESEM with the StdYX coefficients (*p* < 0.05).

To carry out the confirmatory factor analysis, structural equation modelling was used, according to the specific statistical program (Mplus 7) [49]. The adequacy of the factor structure was assessed using the root mean square error of approximation (RMSEA), comparative fit index (CFI), and Tucker–Lewis index (TLI). Figure 3 shows the structural model with the StdYX coefficients.

The fit index was RMSEA (90% CI) = 0.079 (0.076–0.083), χ2 = 2.644, CFI = 0.986 and TLI = 0.985. The ESEM analysis shows that the StdYX coefficients are strong and consistent between the items and between the factors, as detailed in Figure 3. The combination of the fit indices suggests that the ASCIF model demonstrates a good fit to the data. The RMSEA value of 0.079, along with the CFI and TLI values exceeding 0.95, indicates that the model adequately represents the underlying structure of the data collected through the 31 questions on a 5-point Likert scale. The chi-square statistic, while significant, should be interpreted cautiously due to the large sample size (n = 500).

## 4. Discussion

The originality of our research is due to the fact that the validated Awareness Scale on Consumption of Irradiated Foods (ASCIF) [32] was culturally adapted to the Argentine culture and applied to consumers in this country, since in the literature review, no recent research was found on this topic and because we understand that, if the country applies such technology, consumers need to be aware of what they choose, buy and consume. It can be inferred from the theoretical model that, for the Argentine population, the safety of irradiated foods, concepts, and labeling are highly correlated, while awareness shows a low correlation with the other factors, namely the safety of irradiated foods, concepts, and labeling. This result suggests that Argentine consumers are consuming irradiated foods unconsciously, corroborating the result of question 8 (Q8. Consciously consume irradiated foods), which obtained the lowest score in the instrument.

The ASCIF is considered a complex instrument, both because of its subject matter, which is unknown to a large part of the population, and because of its structure, consisting of 4 factors (safety of irradiated foods (S); concepts (C); labeling (L); awareness (A)) distributed in 31 items (S = 15; C = 8; L = 5; A = 3).

The initial translation/retranslation stage of the instrument followed the guidelines of the International Test Commission [19] with adaptations proposed by Borsa et al. [43], increasing the number of participants to five. Most of the items were assessed as unchanged (UC) followed by 21 items classified as little changed (LC), which culminated in the choice of the best ASCIF translation for the Argentine culture, as can be seen in Table 2.

The reliability of the instrument was confirmed by the Cronbach’s alpha index, with a value of 0.988, considered excellent according to Taber [48]. The single-factor, multifactor, and second-order CFA showed strong factor loadings and adequate indices for the instrument, showing signs of validity according to recommendations. The model’s fit indices were above the cut-off points commonly established in the literature: RMSEA values were below 0.08, with an upper limit below 0.10, and CFI and TLI values were above 0.90 [50].

The CFI (comparative fit index) and TLI (Tucker Lewis Index) indices calculate the relative fit of the observed model by comparing it with a base model, whose values above 0.95 indicate a and those above 0.90 indicate an adequate fit [51,52]. The RMSEA (root mean square error of approximation) is also a measure of discrepancy, with results of less than 0.05 expected, but acceptable up to 0.08, although this coefficient penalizes complex models.

It can be seen that the RMSEA value of the instrument showed an acceptable adjustment of the structure, with a result of 0.079. It is worth noting that this coefficient can penalize complex models [6,53], as is the case with the model presented in this study. Finally, in other comparative measures, it was found that the CFI and TLI indices reached acceptable parameters, with a value above 0.90. Thus, the ASCIF model showed signs of validity for its applicability in the Argentine context.

Despite items rarely loading exclusively on one latent factor in multifactorial scales, CFA assumes all indicators/items should load uniquely on their allocated latent dimensions. To address this weakness, exploratory structural equation modelling (ESEM) combines exploratory factor analyses (EFA) and CFA procedures, allowing cross-loadings to occur when assessing hypothesized models [54]. The ESEM of the instrument showed StdYX coefficients with strong correlations, comparable to the results of the CFA (Table 3 and Figure 2). These are important findings of the research that corroborate the evidence of the validity of the ASCIF for the Argentine population.

### 4.1. Potential Applications of the Results

ASCIF can be instrumental in shaping educational curricula, guiding policy decisions and industry practices in food safety and consumer education.

In the field of education, ASCIF can serve as a key tool for developing educational programs aimed at increasing consumer knowledge about food safety and the benefits of irradiated foods. By integrating the results of ASCIF assessments into educational curricula, Argentine educators could adapt their teaching strategies to address knowledge gaps and misconceptions among students and the general public.

From a political point of view, policymakers could take advantage of the data derived from ASCIF assessments to identify demographic groups with lower levels of awareness in order to target them with personalized communication strategies. This targeted approach could improve Argentina’s public health outcomes by ensuring that consumers are better informed about the safety and benefits of irradiated foods, potentially leading to greater acceptance and consumption of such products, consequently improving public health indices with lower outbreaks of foodborne illness. In addition, ASCIF results can guide the development of labeling regulations that clearly communicate the safety and nutritional benefits of irradiated foods to consumers, thus promoting transparency and trust in food systems.

In industrial practice, ASCIF results can be used by food manufacturers and marketers to better understand consumer perceptions and attitudes towards irradiated foods. By analyzing the levels of awareness and safety concerns highlighted in ASCIF, companies can develop marketing strategies that effectively address consumer hesitations and promote the advantages of irradiated products.

In addition, industries can implement training programs for employees that focus on the importance of food safety and the role of irradiation in improving food quality, thus aligning the skills of the workforce with consumer expectations and regulatory standards. This alignment can ultimately lead to better product offerings and greater consumer satisfaction.

### 4.2. Limitations

The research was approved by the ethics committee to be carried out only in the Province of Buenos Aires in Argentina. According to the National Population, Homes and Housing Census carried out in 2022 in Argentina [55], it is understood that this is the region where 38% of the country’s total population is concentrated, with national consumption representativeness, so the research was able to abstract an important extract of the Argentine population. In order to improve the quality of the data, it is suggested that future studies increase the number of participants, expanding the sample to the other Argentine provinces, with the appropriate approval from each of the research ethics committees.

It is important to note that the level of education in Argentina varies significantly between different demographic groups. According to the National Institute of Statistics and Censuses [55], while urban areas, particularly Buenos Aires, have higher levels of education, rural regions generally lag behind. For this reason, the population sample participating in the survey is more educated than the average Argentine population, with many more students than would be expected in a typical population sample.

## 5. Conclusions

Analysis of the results showed that the majority of consumers are unaware of the benefits of irradiated foods. It was found that the instrument met the criteria for evidence of validity and consistency, proving to be an efficient tool for assessing potential challenges and opportunities in the Argentinian market for irradiated foods.

Future research could explore several concrete areas to improve its application in public health campaigns and comparative studies in different countries.

One promising area for future research is the adaptation of the ASCIF tool for use in other cultural contexts. Given that consumer awareness and acceptance of irradiated foods varies significantly between countries, it is crucial to examine how cultural factors influence perceptions of food irradiation. Comparative studies could use ASCIF to assess levels of awareness in different countries, thus identifying specific cultural barriers to the acceptance of irradiated foods and strategies for overcoming them.

Another area of research could focus on the impact of educational campaigns on different demographic groups, such as adults or vulnerable populations, in order to determine the most effective methods for increasing awareness and acceptance of irradiated foods. This could involve longitudinal studies measuring changes in awareness and consumption behaviors before and after the intervention, thus providing information on the effectiveness of educational campaigns.

In addition, it is essential to explore the role of communication strategies in public health campaigns. By integrating the ASCIF tool into these campaigns, researchers can assess how different messaging strategies affect consumer awareness and acceptance of irradiated foods. This can include testing various formats, such as social media, traditional advertising, or community outreach, to determine which methods best suit the target audience.

## Figures and Tables

**Table 1:** Frequency of selected demographic variables of respondents (n = 500).

Demographic Variables	Number of Participants (%)
Gender	
Male	245 (49.0)
Female	255 (51.0)
Age (years)	
<20	25 (5.0)
20–29	99 (19.8)
30–39	142 (28.4)
40–49	115 (23.0)
>50	119 (23.8)
Educational Level	
Elementary school	6 (1.2)
High school	143 (28.6)
Bachelor’s degree	239 (47.8)
Specialization	13 (2.6)
Master’s degree	44 (8.8)
Doctorate degree	55 (11.0)
Average Monthly Family Income (in Minimum Wage—R$)	
<2	95 (19.0)
2–5	120 (24.0)
5–10	118 (23.6)
10–20	89 (17.8)
>20	78 (15.6)
Marital Status	
With partner	299 (59.8)
Without partner	201 (40.2)
Profession	
Public-sector employees	82 (16.4)
Private-sector employees	140 (28.0)
Self employed	51 (10.2)
Student	138 (27.6)
Intern	0 (0.0)
Retired	37 (7.4)
Informal	12 (2.4)
Military	16 (3.2)
Other	24 (4.8)
Nationality	
Argentinean	485 (97.0)
Foreign	15 (3.0)
How many People Live in your House Besides You?	
I live alone	84 (16.8)
Up to 2 people	187 (37.4)
From 2 to 5 people	186 (37.2)
More than 5 people	43 (8.6)
Are you Responsible for Grocery Shopping in Your Home?	
Yes	388 (77.6)
No	112 (22.4)
Region of Residence (Argentina):	
Province of Buenos Aires	455 (91.0)
Another	45 (9.0)

**Table 2:** Semantic equivalence between the original Portuguese version and the Spanish version of ASCIF.

Original ASCIF (Portuguese)	Version 1			Version 2			Final Version
Original Version	Translation 1	Retranslation 1	Version 1 Assessment *	Translation 2	Retranslation 2	Version 2 Assessment *	Comparison Between Versions and Argentine Culture
Q1. Alimento irradiado é diferente de alimento radioativo. (Q1. Irradiated food is different from radioactive food.)	Q1. El alimento irradiado es diferente del alimento radioactivo.	Q1. Alimento irradiado é diferente de alimento radioativo.	UC	Q1. El alimento irradiado no es lo mismo que el alimento radioactivo.	Q1. Alimento irradiado é diferente de alimento radioativo.	UC	Q1. El alimento irradiado es diferente del alimento radioactivo. (Q1. Irradiated food is different from radioactive food.)
Q2. A irradiação de alimentos pode ser utilizada para reduzir a carga microbiana em alimentos. (Q2. Food irradiation can be used to reduce the microbial load on food.)	Q2. La irradiación de alimentos se puede utilizar para reducir la carga microbiana en los alimentos.	Q2. A irradiação de alimentos pode ser utilizada para reduzir a carga microbiana em alimentos.	UC	Q2. La irradiación de alimentos se puede utilizar para reducir la carga microbiana en los alimentos	Q2. A irradiação de alimentos pode ser utilizada para reduzir a carga microbiana em alimentos.	UC	Q2. La irradiación de alimentos se puede utilizar para reducir la carga microbiana en los alimentos. (Q2. Food irradiation can be used to reduce the microbial load on food.)
Q3. A irradiação de alimentos pode ser utilizada para inibir o brotamento de bulbos, raízes e tubérculos. (Q3. The irradiation of food can be used to inhibit the budding of bulbs, roots and tubers.)	Q3. La irradiación de alimentos se puede utilizar para inhibir el brote de bulbos, raíces y tubérculos.	Q3. A irradiação de alimentos pode ser utilizada para inibir o brotamento de bulbos, raízes e tubérculos.	UC	Q3. La irradiación de alimentos se puede utilizar para inhibir la brotación de bulbos, raíces y tubérculos.	Q3. A irradiação de alimentos pode ser utilizada para inibir o brotamento de bulbos, raízes e tubérculos.	UC	Q3. La irradiación de alimentos se puede utilizar para inhibir el brote de bulbos, raíces y tubérculos. (Q3. The irradiation of food can be used to inhibit the budding of bulbs, roots and tubers.)
Q4. A irradiação de alimentos pode ser utilizada para adiar/retardar o amadurecimento de frutas. (Q4. Food irradiation can be used to delay the ripening of fruits.)	Q4. La irradiación de alimentos se puede utilizar para atrasar, retardar la maduración de las frutas.	Q4. A irradiação de alimentos pode ser utilizada para adiar/retardar o amadurecimento de frutas.	UC	Q4. La irradiación de alimentos se puede utilizar para postergar/retrasar la maduración de frutas.	Q4. A irradiação de alimentos pode ser utilizada para adiar/retardar o amadurecimento de frutas.	UC	Q4. La irradiación de alimentos se puede utilizar para atrasar, retardar la maduración de las frutas. (Q4. Food irradiation can be used to delay the ripening of fruits.)
Q5. A dose mínima absorvida pelo alimento irradiado deve ser suficiente para alcançar a finalidade pretendida. (Q5. The minimum absorbed dose by the irradiated food must be sufficient to achieve the intended purpose.)	Q5. La dosis mínima absorbida por el alimento irradiado debe ser suficiente para alcanzar la finalidad pretendida.	Q5. A dose mínima absorvida pelo alimento irradiado deve ser suficiente para alcançar a finalidade pretendida.	UC	Q5. La dosis mínima absorbida por el alimento irradiado debe ser suficiente para alcanzar la finalidad pretendida.	Q5. A dose mínima absorvida pelo alimento irradiado deve ser suficiente para alcançar a finalidade pretendida.	UC	Q5. La dosis mínima absorbida por el alimento irradiado debe ser suficiente para alcanzar la finalidad pretendida. (Q5. The minimum absorbed dose by the irradiated food must be sufficient to achieve the intended purpose.)
Q6. O Brasil autoriza o uso da irradiação de alimentos. (Q6. Brazil authorizes the use of food irradiation.)	Q6. Brasil autoriza el uso de la irradiación de alimentos.	Q6. O Brasil autoriza o uso da irradiação de alimentos.	UC	Q6. Argentina autoriza el uso de la irradiación de alimentos	Q6. A Argentina autoriza o uso da irradiação de alimentos.	LC	Q6. Argentina autoriza el uso de la irradiación de alimentos. (Q6. Argentina authorizes the use of food irradiation.)
Q7. A irradiação de alimentos pode ser utilizada para aumentar a vida útil ou o prazo de validade dos alimentos. (Q7. Food irradiation can be used to increase shelf life.)	Q7. La irradiación de alimentos se puede utilizar para aumentar el plazo de vida útil de los alimentos.	Q7. A irradiação de alimentos pode ser utilizada para aumentar o prazo de vida útil dos alimentos.	LC	Q7. La irradiación de alimentos se puede utilizar para aumentar la vida útil o el plazo de validez de los alimentos.	Q7. A irradiação de alimentos pode ser utilizada para aumentar a vida útil ou o prazo de validade dos alimentos.	UC	Q7. La irradiación de alimentos se puede utilizar para aumentar el plazo de vida útil de los alimentos. (Q7. Food irradiation can be used to increase shelf life.)
Q8. Eu consumo conscientemente alimentos irradiados. (Q8. I consciously consume irradiated food.)	Q8. Yo consumo conscientemente alimentos irradiados.	Q8. Eu consumo conscientemente alimentos irradiados.	UC	Q8. Consumo conscientemente alimentos irradiados.	Q8. Consumo conscientemente alimentos irradiados.	LC	Q8. Yo consumo conscientemente alimentos irradiados. (Q8. I consciously consume irradiated food.)
Q9. Eu consumiria alimentos irradiados. (Q9. I would consume irradiated food.)	Q9. Yo consumiría alimentos irradiados.	Q9. Eu consumiria alimentos irradiados.		Q9. Consumiría alimentos irradiados.	Q9. Consumiria alimentos irradiados.	LC	Q9. Yo consumiría alimentos irradiados. (Q9. I would consume irradiated food.)
Q10. Eu estaria disposto a pagar mais por alimentos irradiados. (Q10. I would be willing to pay more for irradiated food.)	Q10. Yo estaría dispuesto a pagar más por alimentos irradiados.	Q10. Eu estaria disposto a pagar mais por alimentos irradiados.	UC	Q10. Estaría dispuesto a pagar más por los alimentos irradiados.	Q10. Estaria disposto a pagar mais por alimentos irradiados.	LC	Q10. Yo estaría dispuesto a pagar más por alimentos irradiados. (Q10. I would be willing to pay more for irradiated food.)
Q11. Eu incentivaria o consumo de alimentos irradiados. (Q11. I would encourage consumption of irradiated foods.)	Q11. Yo incentivaría el consumo de alimentos irradiados.	Q11. Eu incentivaria o consumo de alimentos irradiados.	UC	Q11. Fomentaría el consumo de alimentos irradiados.	Q11. Incentivaria o consumo de alimentos irradiados.	LC	Q11. Yo incentivaría el consumo de alimentos irradiados. (Q11. I would encourage consumption of irradiated foods.)
Q12. Eu consumiria alimentos irradiados, pois sei que estes não causam danos à saúde. (Q12. I would consume irradiated foods, as I know they do not cause health damage.)	Q12. Yo consumiría alimentos irradiados, porque sé que estos no causan daños a la salud.	Q12. Eu consumiria alimentos irradiados, pois sei que estes não causam danos à saúde.	UC	Q12. Consumiría alimentos irradiados, porque sé que no causan daños a la salud.	Q12. Consumiria alimentos irradiados, pois sei que estes não causam danos à saúde.	LC	Q12. Yo consumiría alimentos irradiados, porque sé que estos no causan daños a la salud. (Q12. I would consume irradiated foods, as I know they do not cause health damage.)
Q13. Eu consumiria alimentos irradiados, pois sei que estes são seguros para o consumo. (Q13. I would consume irradiated food because I know that these are safe for consumption.)	Q13. Yo consumiría alimentos irradiados, porque sé que estos son seguros para el consumo.	Q13. Eu consumiria alimentos irradiados, pois sei que estes são seguros para o consumo.	UC	Q13. Consumiría alimentos irradiados, porque sé que son seguros para el consumo.	Q13. Consumiria alimentos irradiados, pois sei que estes são seguros para o consumo.	LC	Q13. Yo consumiría alimentos irradiados, porque sé que estos son seguros para el consumo. (Q13. I would consume irradiated food because I know that these are safe for consumption.)
Q14. Eu conheço algum alimento irradiado. (Q14. I know some irradiated food.)	Q14. Yo conozco algún alimento irradiado.	Q14. Eu conheço algum alimento irradiado.	UC	Q14. Yo conozco algún alimento irradiado.	Q14. Eu conheço algum alimento irradiado.	UC	Q14. Yo conozco algún alimento irradiado. (Q14. I know some irradiated food.)
Q15. Eu aprovo o consumo de alimentos irradiados. (Q15. I approve of the consumption of irradiated foods.)	Q15. Yo apruebo el consumo de alimentos irradiados.	Q15. Eu aprovo o consumo de alimentos irradiados.	UC	Q15. Apruebo el consumo de alimentos irradiados.	Q15. Aprovo o consumo de alimentos irradiados.	LC	Q15. Yo apruebo el consumo de alimentos irradiados. (Q15. I approve of the consumption of irradiated foods.)
Q16. Eu considero ser necessário fazer campanhas educativas para informar a população sobre a irradiação de alimentos. (Q16. I consider it necessary to carry out educational campaigns to inform the population about the irradiation of food.)	Q16. Yo considero que es necesario hacer campañas educativas para informar a la población sobre la irradiación de alimentos.	Q16. Eu considero ser necessário fazer campanhas educativas para informar a população sobre a irradiação de alimentos.	UC	Q16. Considero que es necesario hacer campañas educativas para informar a la población sobre la irradiación de alimentos.	Q16. Considero ser necessário fazer campanhas educativas para informar a população sobre a irradiação de alimentos.	LC	Q16. Yo considero que es necesario hacer campañas educativas para informar a la población sobre la irradiación de alimentos. (Q16. I consider it necessary to carry out educational campaigns to inform the population about the irradiation of food.)
Q17. Eu conheço a Radura, símbolo utilizado para representar um alimento irradiado. (Q17. I know Radura, the symbol used to represent irradiated food.)	Q17. Yo conozco la Radura: es el símbolo internacional utilizado para representar un alimento irradiado.	Q17. Eu conheço a Radura, símbolo utilizado para representar um alimento irradiado.	UC	Q17. Conozco la Radura, símbolo utilizado para representar los alimentos irradiados.	Q17. Conheço a Radura, símbolo utilizado para representar um alimento irradiado.	LC	Q17. Yo conozco la Radura: es el símbolo internacional utilizado para representar un alimento irradiado. (Q17. I know Radura, the symbol used to represent irradiated food.)
Q18. Todos os alimentos que passam por processo de irradiação deveriam ter essa informação destacada no rótulo do produto. (Q18. All foods that undergo irradiation should have this information highlighted on the product label.)	Q18. Todos los alimentos que pasan por proceso de irradiación deberían tener esa información destacada en la etiqueta del producto.	Q18. Todos os alimentos que passam por processo de irradiação deveriam ter essa informação destacada no rótulo do produto.	UC	Q18. Todos los alimentos que se someten a un proceso de irradiación deberían tener esta información resaltada en la etiqueta del producto.	Q18. Todos os alimentos que passam por processo de irradiação deveriam ter essa informação destacada no rótulo do produto.	UC	Q18. Todos los alimentos que pasan por proceso de irradiación deberían tener esa información destacada en la etiqueta del producto. (Q18. All foods that undergo irradiation should have this information highlighted on the product label.)
Q19. Eu considero que as informações adicionais contidas nos rótulos dos alimentos irradiados são importantes. (Q19. I consider that the additional information contained in the labels of irradiated foods is important.)	Q19. Yo considero que las informaciones adicionales contenidas en las etiquetas de los alimentos irradiados son importantes.	Q19. Eu considero que as informações adicionais contidas nos rótulos dos alimentos irradiados são importantes.	UC	Q19. Considero que es importante la información adicional contenida en las etiquetas de los alimentos irradiados.	Q19. Considero que as informações adicionais contidas nos rótulos dos alimentos irradiados são importantes.	LC	Q19. Yo considero que las informaciones adicionales contenidas en las etiquetas de los alimentos irradiados son importantes. (Q19. I consider that the additional information contained in the labels of irradiated foods is important.)
Q20. Eu considero importante o símbolo da Radura nos rótulos dos alimentos irradiados. (Q20. I consider the symbol of Radura important in the labels of irradiated foods.)	Q20. Yo considero importante el símbolo de la Radura en las etiquetas de los alimentos irradiados.	Q20. Eu considero importante o símbolo da Radura nos rótulos dos alimentos irradiados.	UC	Q20. Considero importante el símbolo de la Radura en las etiquetas de los alimentos irradiados.	Q20. Considero importante o símbolo da Radura nos rótulos dos alimentos irradiados.	LC	Q20. Yo considero importante el símbolo de la Radura en las etiquetas de los alimentos irradiados. (Q20. I consider the symbol of Radura important in the labels of irradiated foods.)
Q21. Eu tenho segurança em comprar um alimento quando leio no rótulo a seguinte informação “alimento tratado por processo de irradiação”. (Q21. I have confidence in buying a food when I read on the label the following information “food treated by irradiation process”.)	Q21. Yo tengo seguridad al comprar un alimento cuando leo en la etiqueta la siguiente información: “alimento tratado por proceso de irradiación”.	Q21. Eu tenho segurança em comprar um alimento quando leio no rótulo a seguinte informação “alimento tratado por processo de irradiação”.	UC	Q21. Me siento seguro al comprar un alimento cuando leo en la etiqueta la siguiente información: “alimento tratado mediante el proceso de irradiación”.	Q21. Sinto-me seguro ao comprar um alimento quando leio no rótulo a seguinte informação: “alimento tratado pelo processo de irradiação”.	LC	Q21. Yo tengo seguridad al comprar un alimento cuando leo en la etiqueta la siguiente información: “Alimento Tratado con Energía Ionizante”. (Q21. I have confidence in buying a food when I read on the label the following information “Food Treated with Ionizing Energy”.)
Q22. O rótulo dos alimentos deveria destacar a informação de alimento irradiado. (Q22. The food label should highlight the information of irradiated food.)	Q22. La etiqueta de los alimentos debería destacar la información de alimento irradiado.	Q22. O rótulo dos alimentos deveria destacar a informação de alimento irradiado.	UC	Q22. Las etiquetas de los alimentos deberían resaltar la información de los alimentos irradiados.	Q22. Os rótulos dos alimentos deveriam destacar a informação de alimento irradiado.	LC	Q22. La etiqueta de los alimentos debería destacar la información de alimento irradiado. (Q22. The food label should highlight the information of irradiated food.)
Q23. Eu compraria alimentos irradiados, pois sei que este processo não torna o alimento radioativo. (Q23. I would buy irradiated food because I know this process does not make the food radioactive.)	Q23. Yo compraría alimentos irradiados, porque sé que este proceso no convierte el alimento en radioactivo.	Q23. Eu compraria alimentos irradiados, pois sei que este processo não torna o alimento radioativo.	UC	Q23. Compraría alimentos irradiados, porque sé que este proceso no los hace radiactivos.	Q23. Compraria alimentos irradiados, pois sei que este processo não torna o alimento radioativo.	LC	Q23. Yo compraría alimentos irradiados, porque sé que este proceso no convierte el alimento en radioactivo. (Q23. I would buy irradiated food because I know this process does not make the food radioactive.)
Q24. Os alimentos irradiados são seguros sob o aspecto microbiológico. (Q24. Irradiated food is microbiologically safe.)	Q24. Los alimentos irradiados son seguros bajo el aspecto microbiológico.	Q24. Os alimentos irradiados são seguros sob o aspecto microbiológico.	UC	Q24. Los alimentos irradiados son seguros desde el punto de vista microbiológico.	Q24. Os alimentos irradiados são seguros do ponto de vista microbiológico.	LC	Q24. Los alimentos irradiados son seguros bajo el aspecto microbiológico. (Q24. Irradiated food is microbiologically safe.)
Q25. Os alimentos irradiados são seguros sob o aspecto nutricional. (Q25. Irradiated foods are nutritionally safe.)	Q25. Los alimentos irradiados son seguros bajo el aspecto nutricional.	Q25. Os alimentos irradiados são seguros sob o aspecto nutricional.	UC	Q25. Los alimentos irradiados son seguros desde el punto de vista nutricional.	Q25. Os alimentos irradiados são seguros do ponto de vista nutricional.	LC	Q25. Los alimentos irradiados son seguros bajo el aspecto nutricional. (Q25. Irradiated foods are nutritionally safe.)
Q26. Eu me sinto seguro quanto ao consumo de alimentos irradiados. (Q26. I feel safe about the consumption of irradiated foods.)	Q26. Yo me siento seguro cuanto al consumo de alimentos irradiados.	Q26. Eu me sinto seguro quanto ao consumo de alimentos irradiados.	UC	Q26. Yo me siento seguro en relación al consumo de alimentos irradiados.	Q26. Eu me sinto seguro quanto ao consumo de alimentos irradiados.	UC	Q26. Yo me siento seguro cuanto al consumo de alimentos irradiados. (Q26. I feel safe about the consumption of irradiated foods.)
Q27. Eu considero que os alimentos irradiados não fazem mal à saúde a curto prazo. (Q27. I consider that irradiated foods are not harmful to health in the short term.)	Q27. Yo considero que los alimentos irradiados no hacen mal a la salud a corto plazo.	Q27. Eu considero que os alimentos irradiados não fazem mal à saúde a curto prazo.	UC	Q27. Considero que los alimentos irradiados no son perjudiciales para la salud a corto plazo.	Q27. Considero que os alimentos irradiados não fazem mal à saúde a curto prazo.	LC	Q27. Yo considero que los alimentos irradiados no hacen mal a la salud a corto plazo. (Q27. I consider that irradiated foods are not harmful to health in the short term.)
Q28. Eu considero que os alimentos irradiados não fazem mal à saúde a médio prazo. (Q28. I consider that irradiated foods are not harmful to health in the medium term.)	Q28. Yo considero que los alimentos irradiados no hacen mal a la salud a medio plazo.	Q28. Eu considero que os alimentos irradiados não fazem mal à saúde a médio prazo.	UC	Q28. Considero que los alimentos irradiados no son perjudiciales para la salud a medio plazo.	Q28. Considero que os alimentos irradiados não fazem mal à saúde a médio prazo.	LC	Q28. Yo considero que los alimentos irradiados no hacen mal a la salud a medio plazo. (Q28. I consider that irradiated foods are not harmful to health in the medium term.)
Q29. Eu considero que os alimentos irradiados não fazem mal à saúde a longo prazo. (Q29. I consider that irradiated foods are not harmful to health in the long term.)	Q29. Yo considero que los alimentos irradiados no hacen mal a la salud a largo plazo.	Q29. Eu considero que os alimentos irradiados não fazem mal à saúde a longo prazo.	UC	Q29. Considero que los alimentos irradiados no son perjudiciales para la salud a largo plazo.	Q29. Considero que os alimentos irradiados não fazem mal à saúde a longo prazo.	LC	Q29. Yo considero que los alimentos irradiados no hacen mal a la salud a largo plazo. (Q29. I consider that irradiated foods are not harmful to health in the long term.)
Q30. Eu considero que os alimentos irradiados não fazem mal à saúde das próximas gerações. (Q30. I consider that irradiated foods are not harmful to the health of future generations.)	Q30. Yo considero que los alimentos irradiados no hacen mal a la salud de las próximas generaciones.	Q30. Eu considero que os alimentos irradiados não fazem mal à saúde das próximas gerações.	UC	Q30. Considero que los alimentos irradiados no son perjudiciales para la salud de las próximas generaciones	Q30. Considero que os alimentos irradiados não fazem mal à saúde das próximas gerações.	LC	Q30. Yo considero que los alimentos irradiados no hacen mal a la salud a largo plazo. (Q30. I consider that irradiated foods are not harmful to the health of future generations.)
Q31. A Organização Mundial da Saúde (OMS) e a Organização das Nações Unidas (FAO/ONU) recomendam a irradiação de alimentos. (Q31. The World Health Organization (WHO) and the United Nations (FAO) recommend the irradiation of food.)	Q31. La Organización Mundial de la Salud (OMS) y la Organización de las Naciones Unidas (FAO/ONU) recomiendan la irradiación de alimentos.	Q31. A Organização Mundial da Saúde (OMS) e a Organização das Nações Unidas (FAO/ONU) recomendam a irradiação de alimentos.	UC	Q31. La Organización Mundial de la Salud (OMS) y la Organización de las Naciones Unidas (FAO/ONU) recomiendan la irradiación de los alimentos.	Q31. A Organização Mundial da Saúde (OMS) e a Organização das Nações Unidas (FAO/ONU) recomendam a irradiação de alimentos.	UC	Q31. La Organización Mundial de la Salud (OMS) y la Organización de las Naciones Unidas (FAO/ONU) recomiendan la irradiación de alimentos. (Q31. The World Health Organization (WHO) and the United Nations (FAO) recommend the irradiation of food.)

* Version Assessment: UC (unchanged); LC (little changed).

**Table 3:** Confirmatory factor analysis (CFA) of the ASCIF using MPlus software.

Items	Means (s.e)	Factor Loadings			
		S ^1^	C ^2^	L ^3^	A ^4^
Q29. I consider that irradiated foods are not harmful to health in the long term. (Q29. Yo considero que los alimentos irradiados no hacen mal a la salud a largo plazo.)	3.090 (0.05)	0.938 (0.005)			
Q11. I would encourage consumption of irradiated foods. (Q11. Yo incentivaría el consumo de alimentos irradiados.)	3.020 (0.05)	0.936 (0.005)			
Q28. I consider that irradiated foods are not harmful to health in the medium term. (Q28. Yo considero que los alimentos irradiados no hacen mal a la salud a medio plazo.)	3.154 (0.05)	0.950 (0.005)			
Q30. I consider that irradiated foods are not harmful to the health of future generations. (Q30. Yo considero que los alimentos irradiados no hacen mal a la salud de las próximas generaciones.)	3.118 (0.05)	0.938 (0.005)			
Q13. I would consume irradiated food because I know that these are safe for consumption. (Q13. Yo consumiría alimentos irradiados, porque sé que estos son seguros para el consumo.)	3.206 (0.05)	0.944 (0.005)			
Q12. I would consume irradiated foods, as I know they do not cause health damage. (Q12. Yo consumiría alimentos irradiados, porque sé que estos no causan daños a la salud.)	3.178 (0.05)	0.953 (0.004)			
Q27. I consider that irradiated foods are not harmful to health in the short term. (Q27. Yo considero que los alimentos irradiados no hacen mal a la salud a corto plazo.)	3.198 (0.05)	0.950 (0.005)			
Q15. I approve of the consumption of irradiated foods. (Q15. Yo apruebo el consumo de alimentos irradiados.)	3.190 (0.05)	0.953 (0.004)			
Q26. I feel safe about the consumption of irradiated foods. (Q26. Yo me siento seguro cuanto al consumo de alimentos irradiados.)	3.094 (0.05)	0.934 (0.005)			
Q10. I would be willing to pay more for irradiated food. (Q10. Yo estaría dispuesto a pagar más por alimentos irradiados.)	2.414 (0.05)	0.855 (0.013)			
Q9. I would consume irradiated food. (Q9. Yo consumiría alimentos irradiados.)	3.040 (0.05)	0.944 (0.005)			
Q21. I have confidence in buying a food when I read on the label the following information “Food Treated with Ionizing Energy”. (Q21. Yo tengo seguridad al comprar un alimento cuando leo en la etiqueta la siguiente información: “Alimento Tratado con Energía Ionizante”.)	3.140 (0.05)	0.914 (0.007)			
Q23. I would buy irradiated food because I know this process does not make the food radioactive. (Q23. Yo compraría alimentos irradiados, porque sé que este proceso no convierte el alimento en radioactivo.)	3.322 (0.05)	0.954 (0.004)			
Q25. Irradiated foods are nutritionally safe. (Q25. Los alimentos irradiados son seguros bajo el aspecto nutricional.)	3.156 (0.05)	0.928 (0.006)			
Q31. The World Health Organization (WHO) and the United Nations (FAO) recommend the irradiation of food. (Q31. La Organización Mundial de la Salud (OMS) y la Organización de las Naciones Unidas (FAO/ONU) recomiendan la irradiación de alimentos.)	3.038 (0.06)	0.940 (0.006)			
Q2. Food irradiation can be used to reduce the microbial load on food. (Q2. La irradiación de alimentos se puede utilizar para reducir la carga microbiana en los alimentos.)	3.304 (0.05)		0.941 (0.005)		
Q7. Food irradiation can be used to increase shelf life. (Q7. La irradiación de alimentos se puede utilizar para aumentar el plazo de vida útil de los alimentos.)	3.340 (0.05)		0.938 (0.006)		
Q3. The irradiation of food can be used to inhibit the budding of bulbs, roots, and tubers. (Q3. La irradiación de alimentos se puede utilizar para inhibir el brote de bulbos, raíces y tubérculos.)	3.264 (0.05)		0.925 (0.007)		
Q5. The minimum absorbed dose by the irradiated food must be sufficient to achieve the intended purpose. (Q5. La dosis mínima absorbida por el alimento irradiado debe ser suficiente para alcanzar la finalidad pretendida.)	3.386 (0.05)		0.895 (0.009)		
Q6. Argentina authorizes the use of food irradiation. (Q6. Argentina autoriza el uso de la irradiación de alimentos.)	3.066 (0.05)		0.895 (0.009)		
Q24. Irradiated food is microbiologically safe. (Q24. Los alimentos irradiados son seguros bajo el aspecto microbiológico.)	3.278 (0.05)		0.947 (0.005)		
Q4. Food irradiation can be used to delay the ripening of fruits. (Q4. La irradiación de alimentos se puede utilizar para atrasar, retardar la maduración de las frutas.)	3.244 (0.04)		0.933 (0.006)		
Q1. Irradiated food is different from radioactive food. (Q1. El alimento irradiado es diferente del alimento radioactivo.)	3.302 (0.05)		0.848 (0.006)		
Q18. All foods that undergo irradiation should have this information highlighted on the product label. (Q18. Todos los alimentos que pasan por proceso de irradiación deberían tener esa información destacada en la etiqueta del producto.)	3.506 (0.05)			0.911 (0.009)	
Q20. I consider the symbol of Radura important in the labels of irradiated foods. (Q20. Yo considero importante el símbolo de la Radura en las etiquetas de los alimentos irradiados.)	3.420 (0.05)			0.950 (0.007)	
Q22. The food label should highlight the information of irradiated food. (Q22. La etiqueta de los alimentos debería destacar la información de alimento irradiado.)	3.382 (0.05)			0.956 (0.006)	
Q19. I consider that the additional information contained in the labels of irradiated foods is important. (Q19. Yo considero que las informaciones adicionales contenidas en las etiquetas de los alimentos irradiados son importantes.)	3.706 (0.04)			0.856 (0.013)	
Q16. I consider it necessary to carry out educational campaigns to inform the population about the irradiation of food. (Q16. Yo considero que es necesario hacer campañas educativas para informar a la población sobre la irradiación de alimentos.)	3.884 (0.04)			0.837 (0.017)	
Q14. I know some irradiated food. (Q14. Yo conozco algún alimento irradiado.)	3.212 (0.05)				0.859 (0.012)
Q17. I know Radura, the symbol used to represent irradiated food. (Q17. Yo conozco la Radura: es el símbolo internacional utilizado para representar un alimento irradiado.)	3.248 (0.05)				0.832 (0.013)
Q8. I consciously consume irradiated food. (Q8. Yo consumo conscientemente alimentos irradiados.)	2.396 (0.05)				0.820 (0.017)

^1^ Safety of irradiated foods (S). ^2^ Concepts (C). ^3^ Labeling (L). ^4^ Awareness (A).

**Table 4:** Component correlation matrix.

Component	S	C	L	A
S	1.000			
C	0.711	1.000		
L	0.627	0.438	1.000	
A	−0.738	−0.650	−0.476	1.000

## Data Availability

The raw data supporting the conclusions of this article will be made available by the authors on request.
